# Examining the comorbidity network of Internet addiction and depression: the role of effortful control on their bridge symptoms in adolescents

**DOI:** 10.3389/fpsyt.2025.1493888

**Published:** 2025-02-13

**Authors:** Tomoya Hirota, Masaki Adachi, Rei Monden, Hiroyuki Mori, Michio Takahashi, Kazuhiko Nakamura

**Affiliations:** ^1^ Department of Psychiatry and Behavioral Sciences, Weill Institute for Neurosciences, University of California, San Francisco, San Francisco, CA, United States; ^2^ Department of Neuropsychiatry, Graduate School of Medicine, Hirosaki University, Hirosaki, Japan; ^3^ Faculty of Psychology, Meiji Gakuin University, Tokyo, Japan; ^4^ Research Center for Child Mental Development, Graduate School of Medicine, Hirosaki University, Hirosaki, Japan; ^5^ Graduate School of Advanced Science and Engineering, Hiroshima University, Higashi-Hiroshima, Japan; ^6^ Department of Psychology, Faculty of Humanities, Saitama Gakuen University, Kawaguchi, Japan; ^7^ Smart-Aging Research Center, Tohoku University, Sendai, Japan

**Keywords:** bridge symptoms, comorbidity, Internet addiction, effortful control, network analysis

## Abstract

**Background and aims:**

Internet addiction (IA) and depression commonly co-occur in adolescents, yet the mechanisms underlying their comorbidity remain unclear. This study aims to elucidate the comorbidity mechanism through network analysis, identifying bridge symptoms linking IA and depression, and exploring sex differences. Additionally, the study examines the association between effortful control (EC) and bridge symptoms, providing insights for interventions.

**Methods:**

A school-based survey was conducted among 7th to 9th-grade students in Japan. Participants completed questionnaires assessing IA (measured by the Young Diagnostic Questionnaire), depression (measured by the Patient Health Questionnaire for Adolescents), and EC (measured by the Early Adolescent Temperament Questionnaire). Network analysis was employed to identify bridge symptoms and examine their association with EC. Bootstrapping for network analysis was conducted to assess network accuracy and stability as well as sex differences in the network structures.

**Results:**

Among the 4,111 students approached, 3,909 (1,904 male and 2,005 female) students filled out the survey. Bridge symptoms such as “Escape” (from the IA cluster) and “Concentration” (from the depression cluster) were found important in both male and female students. Our analysis also revealed differences in the importance of the bridge symptoms across males and females with “Psychomotor” symptoms (from the depression cluster) predominantly in males and “Feeling Guilty” (from the depression cluster) and “Functional impairment” (from the IA cluster) predominantly in females. EC showed a notable negative association with “Concentration”, suggesting important relationships between the transdiagnostic factor and bridge symptoms in understanding the comorbid conditions. The network comparison test did not reveal significant differences in the network structures across sexes.

**Discussion and conclusions:**

The study revealed differences in bridge symptoms linking IA and depression between male and female students. Our findings provide valuable insights for understanding the comorbidity mechanisms of IA and depression in adolescents. Further research using a longitudinal study design is warranted to identify the directionality between EC and bridge symptoms.

## Introduction

Although not officially identified as a mental disorder in the Diagnostic and Statistical Manual of Mental Disorders-Fifth Edition, Internet addiction (IA) has garnered significant attention in behavioral addiction research due to its profound impact on the health and well-being of affected individuals (1). IA is strongly associated with mental health disorders (2, 3), leading to increased disease burden (4), as evidenced by disruptions in daily life and more severe impairment in social functioning (5).

Network analysis has emerged as a popular method for examining comorbidity both at symptom levels and domain levels (6, 7). This approach diverges from traditional methods that treat IA and depression within a latent variable model, potentially obscuring meaningful associations existing between individual symptoms given that both IA and depression are complex human behavior phenomena and composed of heterogeneous observable signs and symptoms. In the network model, IA and depression are conceptualized as complex networks of mutually reinforcing symptoms (8). This framework facilitates the identification of the overall network structure of disorders by quantifying associations among observable symptoms and can uncover symptoms linking different disorders, “bridge symptoms” (9), which may contribute to understand the comorbidity mechanism and developing effective interventions (10, 11).

Previous studies conducted in China using a network approach revealed several bridge symptoms of the IA-depression comorbidity network in adolescents and young adults, such as feelings of guilt, using the Internet to escape from problems, and anticipating future online activities (12, 13). However, there is a dearth of research examining sex differences in the comorbidity network of these conditions, despite documented sex differences in the symptomatology of IA (14) and depression (15) with disparities reported to emerge in early adolescence (16). Identifying differences in the bridge symptoms across males and females in adolescent populations can deepen our understanding of the IA-depression comorbidity mechanism and aid in developing sex-specific interventions.

Additionally, research underscores the pivotal role of transdiagnostic factors in mental health comorbidity (17). Effortful control (EC), a construct originating from temperamental research (18), is synonymous with self-regulation and comprises three subdomains: activation control, inhibitory control, and attention control. EC is hypothesized as a critical locus for the influence on cognition, emotion, and behavior, and impulse control (19), and thus poor EC is closely linked with psychopathology, including internalizing disorders and behavioral addiction (20). Given the complexities of treating psychiatric comorbidities (21), further understanding the association between transdiagnostic factors, such as EC/self-regulation, and individual symptoms of IA and depression, particularly bridge symptoms, is crucial for developing future interventions.

In this study, we aimed to 1) identify differences in bridge symptoms across males and females in the IA-depression comorbidity network using network analysis in a general population sample of middle school students and 2) explore the association between EC and bridge symptoms and other symptoms of IA and depression.

## Methods

### Population and study settings

This study constitutes a secondary data analysis of information derived from a school-based survey conducted in Hirosaki, Japan, in 2019. We distributed letters containing pertinent information to the parents or guardians of all 7th to 9th-grade students (equivalent to 12-15 years of age) enrolled in national or public schools within Hirosaki city. The number of middle schools approached was 17, with 4111 students (1995 boys and 2116 girls), which covered 98.6% of all middle school students in the city. Students whose parents or guardians did not provide consent for their participation were excluded from the study. Subsequently, the students were briefed about the survey’s purpose and proceeded to complete the questionnaire within their respective classrooms.

### Measures

For the assessment of IA, we employed the Young Diagnostic Questionnaire (YDQ) (22) (**Table 1**). The YDQ comprises eight items and is structured based on the criteria for pathological gambling outlined in the Diagnostic Statistical Manual of Mental Disorders, fourth edition (DSM-IV). Respondents provide binary responses (“yes” or “no”) to each item, facilitating the handling of Internet use data as binary information.

**Table 1:** Individual items of the measurements used in the study.

Item	Node name	Centrality name
PHQ9-A		
Feeling down, depressed, irritable, or hopeless	PHQ1	Mood
Little interest or pleasure in doing things	PHQ2	Anhedonia
Trouble falling or staying asleep, or sleeping too much	PHQ3	Insomnia
Feeling tired or having little energy	PHQ4	Fatigue
Poor appetite or overeating	PHQ5	Appetite
Feeling bad about yourself	PHQ6	Feeling guilty
Trouble concentrating on things	PHQ7	Concentration
Moving or speaking so slowly or fidgety or restless	PHQ8	Psychomotor
Thoughts that you would be better off dead, or of hurting yourself	PHQ9	Suicide
YDQ		
Preoccupation with the Internet	YDQ1	Preoccupation
Increased amounts of time on the Internet needed to achieve satisfaction	YDQ2	Tolerance
Repeated failure to control Internet use	YDQ3	Failure to control
Restlessness or irritability when attempting to cut down or stop Internet use	YDQ4	Withdrawal
Staying online longer than originally intended	YDQ5	Increased time
Impaired social, academic, or work functioning	YDQ6	Functional impairment
Lies to others to conceal the extent of involvement with Internet use	YDQ7	Lies
Use of Internet to escape from problems or relieve dysphoric mood	YDQ8	Escape

Depression symptoms were evaluated using the Patient Health Questionnaire for Adolescents (PHQ-A), as outlined in **Table 1**. The PHQ-A is a self-reporting questionnaire adapted from the PHQ-9, which is widely recognized as one of the most frequently utilized depression screening tools (23). Respondents provide responses on a four-point Likert-type scale, with each item scored from 0 (not at all) to 3 (nearly every day). Participants were prompted to reflect on their feelings over the past two weeks, addressing symptoms such as “Little interest or pleasure in doing things” and “Feeling down, depressed, or hopeless.” The total score ranges from 0 to 27, with higher scores indicating a more severe level of depression.

Effortful control (EC) was assessed using the subscale score of the Early Adolescent Temperament Questionnaire Revised (EATQ-R) (24). EC encompasses three components: activation control, inhibitory control, and attention control. Activation control comprises five items related to the ability to activate appropriate responses when tasks demand changes. Inhibitory control consists of five items assessing the ability to inhibit maladaptive behavioral responses, while attention control comprises six items evaluating the ability to maintain or shift attentional focus. Each item was rated on a scale from 1 (“Hardly true”) to 5 (“Mostly true”). In our study, we utilized the total score of EC, which is the sum of scores from all 16 items, treated as continuous data.

### Statistical analysis

#### Network estimation

In the present study, we constructed a regularized partial correlation network using the mixed graphical model (MGM) to estimate the network structure of binary data (8 symptoms of IA) and non-binary data (9 symptoms of depression). In the network model, nodes represent individual items, and relationships between nodes are defined as edges. Edges are understood as partial associations between two nodes while controlling for all other nodes in the network, and their thickness or weights reflect the strength of association (an estimation of partial correlation coefficients). The lack of edge between two nodes means conditional independence relationships among the nodes. The network was estimated and visualized using the R-package ‘mgm’ in the statistical program ‘R version 4.3.2’ (25). To create a more parsimonious network and minimize the likelihood of type-I errors, the graphical LASSO, reducing the edges by shrinking the smallest edges exactly to zero in the R package ‘qgraph’, was used. This process of regularization is coupled with best-fit model selection, by minimizing an information criterion, in this case, the Extended Bayesian Information Criterion (EBIC) (26). We used the Fruchterman-Reingold algorithm for network visualization, a force-directed algorithm that encourages closely related nodes to be plotted near each other (27) and set “lambdaSel = EBIC” for model selection.

#### Bridge symptoms and bridge centrality

The bridge function in the R package ‘networktools’ (version 1.4.0) was used (11) to identify potential bridging symptoms (i.e., symptoms that are influential in connecting different clusters), which were selected based on the bridge expected influence (EI). Bridge EI is a centrality index quantifying how strongly and directly a symptom node is associated with all other symptoms in the network and is generally presented as standardized Z-scores, with higher values reflecting greater overall importance of a symptom to the network, and symptoms that are high in bridge EI are important for connecting broader clusters of symptoms.

#### Network accuracy and stability

To investigate the reliability and robustness of the study results, we assessed the accuracy and stability of network edges and centrality indices using the R package ‘bootnet’ (28). More specifically, using nonparametric bootstrap methods, we estimated network stability as follows: 1) constructed a 95% bootstrapped confidence interval around the regularized edge weights, 2) computed an edge-weight difference test as well as centrality difference test, and 3) estimated the correlation stability coefficient of centrality indices (via a case-dropping bootstrap procedure). Centrality indices were considered strongly stable if the values of the correlation stability coefficient were over 0.5, while values below 0.25 indicated inadequate stability (29).

#### Network comparison between female and male students

Networks from different groups can be compared in the following three aspects: 1) edge strength invariance and 2) global connectivity invariance (30). The first aspect pertains to the difference in the strength of a specific edge of interest if statistically significant differences in the edge strengths are identified. The second aspect is related to the overall level of connectivity (i.e. whether symptoms are connected to the same degree across groups or not). Networks for the male student group and the female student group were re-estimated for both of these groups and compared using the ‘NetworkComparisonTest (NCT)’ package (30) which tests for the above-mentioned aspects using non-parametric permutation tests (1000 random permutations were used in this study).

#### Investigation of the association between psychopathology and effortful control in the network model

To clarify how psychopathology (depression and IA) is associated with EC, we first added a total score of EC (i.e. sum of scores of three subdomains, including activation control, inhibitory control, and attention) to the dataset of psychopathology and estimated the network. We then added each of these subdomain scores to the dataset of psychopathology and repeated the same procedure.

### Ethical issues

The protocol of the study received approval from the Committee on Medical Ethics of Hirosaki University (IRB# 2015-055), and the present study was conducted in adherence to the ethical standards outlined in the 1964 Declaration of Helsinki and its subsequent amendments.

## Results

### Demographics and characteristics of the study participants

Among 4,111 students approached, 3,909 (1,904 male and 2,005 female) students filled out the survey. Demographics and characteristics of these students are summarized in **Table 2**.

**Table 2:** Characteristics of study participants.

	Male (N = 1,904)	Female (N = 2,005)	
Grade			
7	645	664	
8	646	652	
9	613	669	
PHQ-A total*	4.1	5	*p* <.05, Cohen’s *d* = 0.18
YDQ total*	1.4	1.6	*p* <.05, Cohen’*s d* = 0.10
EC total*	86.9	87.8	

*Mean total scores.

PHQ-A, Patient Health Questionnaire-9 modified for adolescents; YDQ, Young Diagnostic Questionnaire; EC, Effortful control.

### Bridge symptoms, and bridge centrality in the network of IA and depression

**Figure 1:**
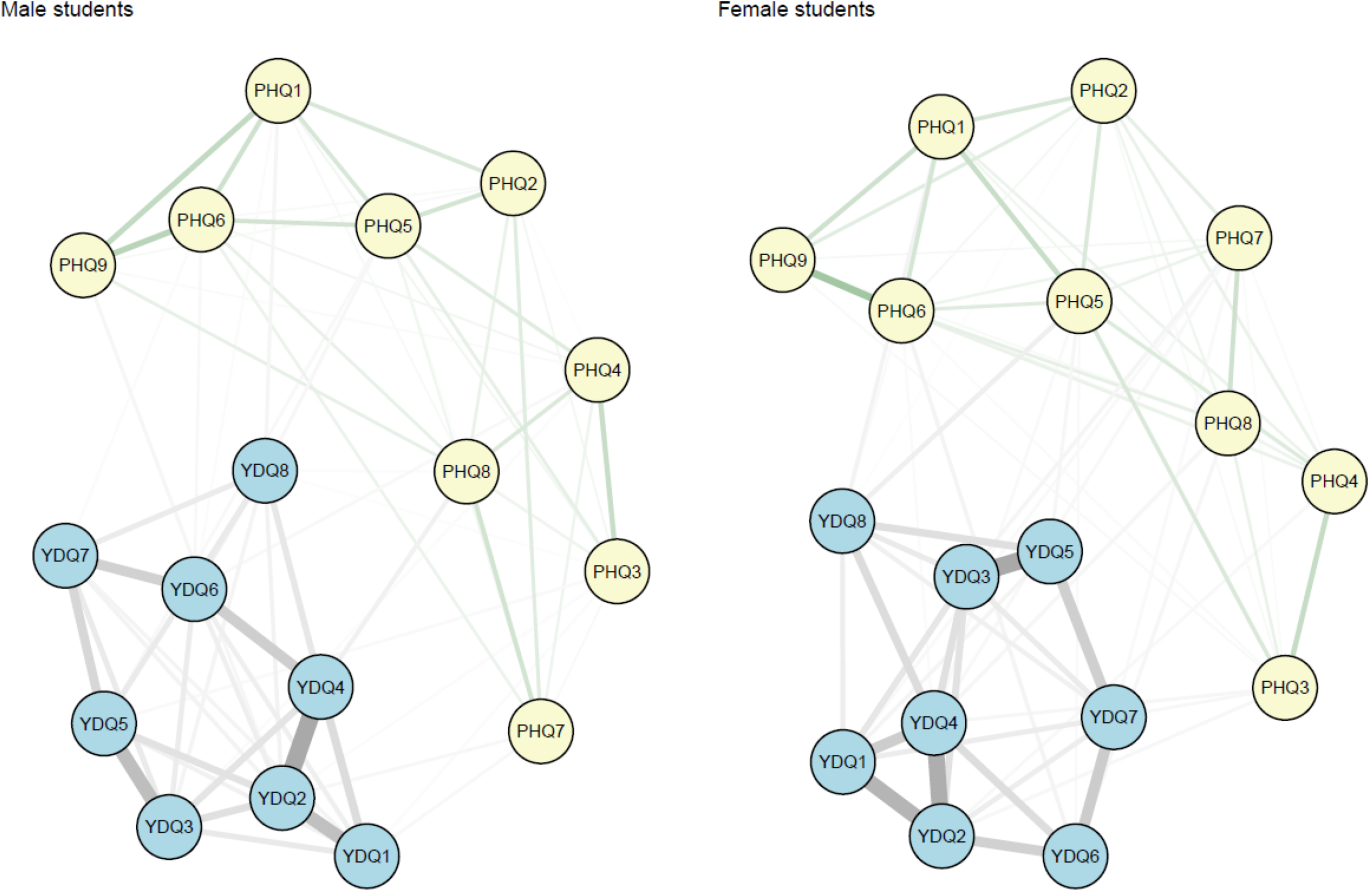
Network structures of Internet addiction and depression symptoms by sex. The comorbidity network structure of internet addiction and depression. In a network, variables are represented by nodes, and relationships between variables/nodes are represented by edges. Edges represent regularized partial relationships. Green edges represent positive associations. Thicker edges between symptoms denote stronger associations. Grey edges relate to edges connecting a pair of variables of at least one variable is a categorical variable with more than two parameters. In this case, the dependency, parameters behind edge, is parameterized by more than one parameter, and therefore there is no notion of a linear relationship. PHQ1, Mood; PHQ2, Anhedonia; PHQ3, Insomnia; PHQ4, Fatigue; PHQ5, Appetite; PHQ6, Feeling guilty; PHQ7, Concentration; PHQ8, Psychomotor; PHQ9, Suicide; YDQ1, Preoccupation; YDQ2, Tolerance; YDQ3, Failure to control; YDQ4, Withdrawal; YDQ5, Increased time; YDQ6, Functional impairment; YDQ7, Lies; YDQ8, Escape.

**Figure 1** illustrates the network structures depicting the relationships among symptoms of IA and depression in study participants. Each edge strength among items in the network of depression and IA is listed in **Supplementary Material 1**. Within the network, “escape” (YDQ8: “*Use of Internet to escape from problems or relieve dysphoric mood*”), “concentration difficulty” (PHQ7: “*Trouble concentrating on things*”), and “psychomotor agitation or slowness” (PHQ8: “*Moving or* sp*eaking so slowly or fidgety or restless*”) identified as bridge symptoms among male students, exhibiting high values of bridge expected influence index (**Table 3**). Conversely, in female students, “functional impairment” (YDQ6: “*Impaired social, academic, or work functioning*”), “escape” (YDQ8: “*Use of Internet to escape from problems or relieve dysphoric mood*”), “feeling guilty” (PHQ6: “*Feeling bad about yourself*”), and “concentration difficulty” (PHQ7: “*Trouble concentrating on things*”) were identified as bridge symptoms owing to their high values of bridge expected influence index. Detailed values of the bridge expected influence within these networks are provided in **Table 3**.

**Table 3:** Bridge expected influence values.

	Male students	Female students
PHQ1: Mood	0.11	0.15
PHQ2: Anhedonia	0.00	0.00
PHQ3: Insomnia	0.22	0.00
PHQ4: Fatigue	0.00	0.00
PHQ5: Appetite	0.11	0.18
PHQ6: Guilty feeling	0.00	0.34
PHQ7: Concentration	0.43	0.27
PHQ8: Psychomotor	0.28	0.13
PHQ9: Suicide	0.00	0.00
YDQ1: Preoccupation	0.14	0.18
YDQ2: Tolerance	0.22	0.00
YDQ3: Failure to control	0.12	0.12
YDQ4: Withdrawal	0.18	0.11
YDQ5: Increased time	0.18	0.00
YDQ6: Functional impairment	0.00	0.30
YDQ7: Lies	0.00	0.05
YDQ8: Escape	0.30	0.32

Bridge expected influence values are presented as standardized Z-scores, with higher values reflecting greater overall importance of a symptom to the network.

### Network comparison between male and female students

The network comparison demonstrated that no edges were statistically different (*p* = 0.91) between the male and female students, indicating similarity in network structures across sexes. Additionally, the comparison test revealed that the global edge strengths did not differ significantly between the two groups (*p* = 0.59).

### Network accuracy and stability

The bootstrap analysis of edge weights in the network analysis of depression and IA symptoms (**Supplementary Material 2**) revealed overlapping 95% confidence intervals for many edges in both male and female students. Furthermore, there were minimal significant differences observed among the strong edges (**Supplementary Material 3**). These results suggest that the majority of edges do not exhibit significant differences, emphasizing the need for cautious interpretation of the ranking of edge weights. The difference test of the node centrality (expected influence) revealed statistically significant differences between “Concentration” and “Anhedonia”, “Concentration” and “Lies”, “Escape” and “Lies” in male students and between “Escape” and “Anhedonia”, “Escape” and “Increased time”, and “Escape” and “Lies” in female students (**Supplementary Material 4**). Regarding centrality indices stability, as demonstrated in **Supplementary Material 5**, the bridge expected influence showed satisfactory stability for both male and female students, with values of 0.60 and 0.59, respectively. Bootstrapped 95% confidence interval (CI) of bridge EI is also listed in **Supplementary Material 6**, revealing wide 95% CI in both male and female students.

### Association between effortful control, IA, and depression

**Figure 2:**
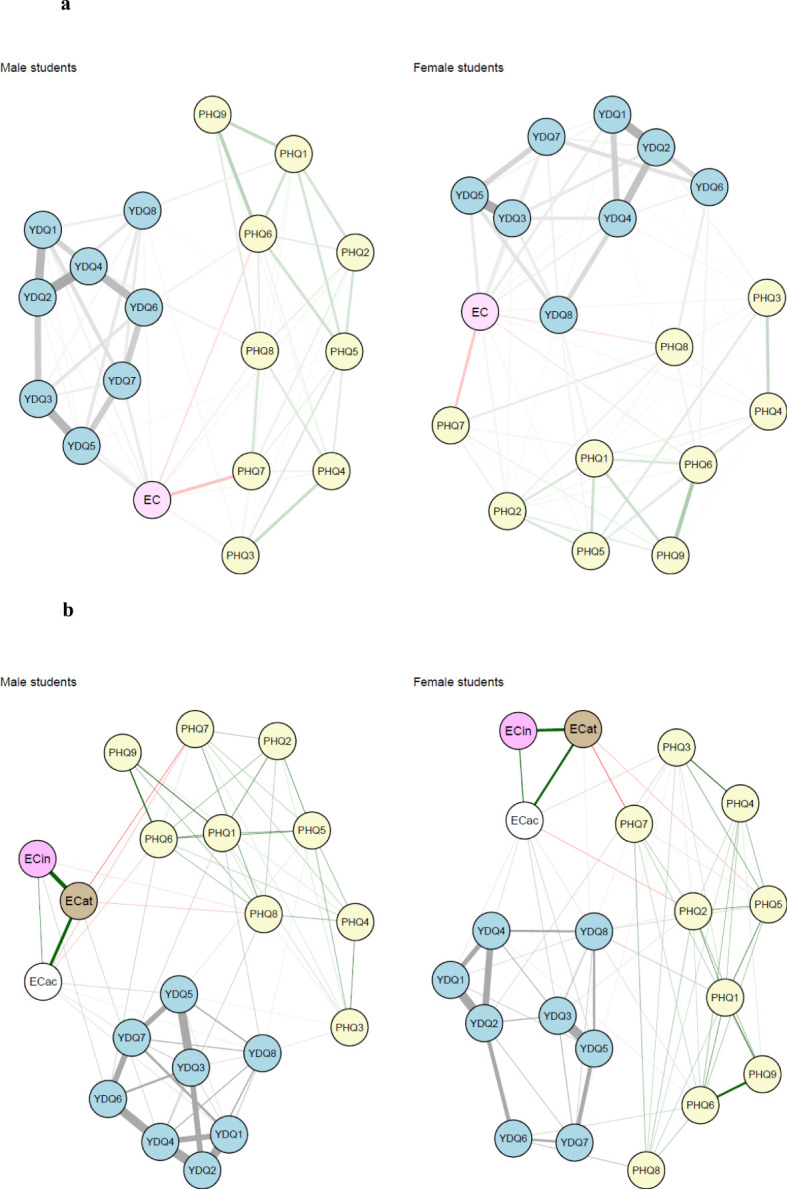
Network structures of Internet addiction and depression symptoms by sex after effortful control (EC) being added [**(A)** with the total score of EC, **(B)** with three different subdomain scores of EC]. **(A)** The comorbidity network structure of internet addiction and depression with the total score of effortful control being added. In a network, variables are represented by nodes, and relationships between variables/nodes are represented by edges. Edges represent regularized partial relationships, where green and red edges indicate positive and negative associations, respectively. Thicker edges between symptoms denote stronger associations. Grey edges relate to edges connecting a pair of variables of at least one variable is a categorical variable with more than two parameters. In this case, the dependency, parameters behind edge, is parameterized by more than one parameter, and therefore there is no notion of a linear relationship. PHQ1, Mood; PHQ2, Anhedonia; PHQ3, Insomnia; PHQ4, Fatigue; PHQ5, Appetite; PHQ6, Feeling guilty; PHQ7, Concentration; PHQ8, Psychomotor; PHQ9, Suicide; YDQ1, Preoccupation; YDQ2, Tolerance; YDQ3, Failure to control; YDQ4, Withdrawal; YDQ5, Increased time; YDQ6, Functional impairment; YDQ7, Lies; YDQ8, Escape; EC, Effortful control. **(B)** The comorbidity network structure of internet addiction and depression with the total score of effortful control being added. In a network, variables are represented by nodes, and relationships between variables/nodes are represented by edges. Edges represent regularized partial relationships, where green and red edges indicate positive and negative associations, respectively. Thicker edges between symptoms denote stronger associations. Grey edges relate to edges connecting a pair of variables of at least one variable is a categorical variable with more than two parameters. In this case, the dependency, parameters behind edge, is parameterized by more than one parameter, and therefore there is no notion of a linear relationship. PHQ1, Mood; PHQ2, Anhedonia; PHQ3, Insomnia; PHQ4, Fatigue; PHQ5, Appetite; PHQ6, Feeling guilty; PHQ7, Concentration; PHQ8, Psychomotor, PHQ9, Suicide; YDQ1, Preoccupation; YDQ2, Tolerance; YDQ3, Failure to control; YDQ4, Withdrawal; YDQ5, Increased time; YDQ6, Functional impairment; YDQ7, Lies; YDQ8, Escape; ECac, Activation control (EC subdomain); ECat, Attention (EC subdomain); ECin, Inhibitory control (EC subdomain).

**Figures 2A, B** illustrate the network structures subsequent to the inclusion of the EC total score and EC subdomain scores (activation control, inhibitory control, and attention) as variables in the IA and depression network, respectively. Among male students, the most pronounced negative association was observed between the total EC score and the concentration symptom (PHQ7: *“Trouble concentrating on things”*) with an edge strength of - 0.21. In addition to the concentration symptom, the negative association with EC was notably stronger in three IA symptoms among male students. Specifically, the association was stronger in IA symptoms YDQ3: *“Repeated failure to control Internet use”*, YDQ5: *“Staying online longer than originally intended”*, YDQ7: *“Lies to others to conceal the extent of involvement with Internet use*, compared to other IA or depressive symptoms. Among the three identified bridge symptoms in male students (YDQ8, PHQ7, and PHQ8), only one bridge symptom (PHQ7: *“Trouble concentrating on things”*) exhibited a noticeable association with effortful control (EC). The association between psychopathology and subdomain scores of EC was overall small with the highest value being 0.13 (YDQ5 and activation control, YDQ6 and inhibitory control, PHQ7 and attention, and YDQ4 and attention). The values of the association between PHQ7, one of the bridge symptoms identified in this study, and EC subdomains were 0.13 (with attention), 0.06 (with activation control), and 0 (with inhibitory control). All edge strengths between EC and Internet Addiction (IA) or depressive symptoms are listed in **Supplementary Material 7**.

In female students, the strongest negative association between EC was observed with the “increased time” (YDQ5: *“Staying online longer than originally intended”*), with an edge strength of 0.23. Additionally, EC exhibited stronger associations with other IA symptoms, including YDQ1: *“Preoccupation with the Internet”*, YDQ3: “*Repeated failure to control Internet use”*, and YDQ7: *“Lies to others to conceal the extent of involvement with Internet use”*, as well as one depressive symptom (PHQ7: *“Trouble concentrating on things”*) compared to other symptoms. Among four identified bridge symptoms (PHQ6, PHQ7, YDQ6, and YDQ8), only one bridge symptom (PHQ7: *“Trouble concentrating on things”*) showed a notable association with EC in female students (**Supplementary Material 5**). The values of edge strengths between psychopathology and EC subdomains were overall small in female students except for those between PHQ7 and attention subdomain (0.20), YDQ3 and activation control subdomain (0.14), and YDQ5 and activation control subdomain (0.13).

The network comparison demonstrated that no edges were statistically different between the male and female students both at the total EC score level (*p* = 0.10) and the subdomain EC level (*p* = 0.07), indicating similarity in network structures across sexes. Additionally, the network comparison test revealed that the global edge strengths did not differ significantly between the two groups either at the total EC score level (*p* = 0.30) or at the subdomain EC level (*p* = 0.19).

## Discussion

Recent applications of the network model to mental health disorders, wherein disorders are conceptualized as arising from the interactions of individual symptoms mutually influencing each other, emphasize the importance of further understanding the role of bridge symptoms in comorbidities (6, 31).

In our study, we investigated bridge symptoms of IA and depression and their sex differences among middle school (7th – 9th grade) students using network analysis. We found “Escape” (an IA symptom) and “Concentration” (a depressive symptom) as bridge symptoms in both male and female middle school students. Few studies have used network analysis to elucidate bridge symptoms of IA and depression. In one Chinese study, researchers identified two bridge symptoms (“Feeling bad about yourself, or that you are a failure or have let yourself or your family down (Feeling guilty)” and “Using the internet as a way of escaping from problems or of relieving a dysphoric mood (Escape)”) in college students (13). In another study in China, researchers identified three bridge symptoms (“Anticipation for future online activities”, “Fear that life is boring and empty without the Internet”, and “Spending more time online over going out with others”) in 7th-12th grade students (12). Differences in participant age (middle school students in our study vs. older adolescents and young adults in previous studies), measures used, and potential effects of the COVID-19 pandemic on participants’ behavior and emotions (pre-pandemic data in our study vs. post-pandemic data in previous studies) likely contributed to variations in identified bridge symptoms. However, it is noteworthy that two bridge symptoms (“Guilty feeling” and “Escape”) were replicated in our study.

Our study primarily examined the relationship between IA and depression through a network analysis perspective. However, our findings may extend to the association between other behavioral addictions and depression, as similar bridge symptoms have been identified in previous research. For instance, one study identified “Escape” as a bridge symptom within the network of internet gaming disorder and depression in children and adolescents (32). Similarly, another study reported the same bridge symptoms “Escape” and “Concentration problems” as bridge symptoms in the network of problematic smartphone use/smartphone addiction and depression (33). These findings, together with our own, suggest the possibility of a unified mechanism underlying the comorbidity between behavioral addictions and depression, irrespective of the specific addictive activity.

Additionally, our study revealed sex differences in bridge symptoms between IA and depression. “Psychomotor” (a depressive symptom) was notable as a bridge symptom only in male students, and “Guilty feeling” (a depressive symptom) and “Functional impairment” (an IA symptom) were identified as bridge symptoms only in female students, suggesting different roles of bridge symptoms between males and females in the formation of comorbid IA and depression networks in adolescence. Sex differences in bridge symptoms of IA and depression found in our study may be explained by differences in preferred engagement styles in online activities. While males are more prone to Internet gaming addiction, potentially contributing to psychomotor agitation or restlessness (“Psychomotor” symptom, a bridge symptom only in male students), females tend to use social media more frequently, possibly affecting self-esteem and leading to guilty feelings (PHQ6: “Feeling bad about yourself”) (34).

In the present study, we investigated the role of effortful control (EC) in the networks of IA and depression symptoms to clarify its association with these two conditions at the symptom level. Contrary to our hypothesis that EC would be strongly associated with most bridge symptoms, a noticeable association with EC was found only in one bridge symptom (PHQ7: *“Trouble concentrating on things”*) both in male students and female students, among the three and four identified bridge symptoms, respectively. However, the negative association between the total score of EC and the concentration symptom was strongest in male students and the second strongest in female students. Longitudinal research is needed to identify the directionality between EC and bridge symptoms. Such research can also allow us to explore how the association between EC and bridge symptoms of IA-depression networks changes with interventions aimed at improving EC and accelerating the development of effective interventions for these comorbid psychiatric conditions given the effectiveness of universal interventions to improve self-regulation in children and adolescents, a concept interchangeably used with EC, on mental health and behavioral problems (35). However, caution is required in interpreting our findings. Given that attention subdomain of EC mostly accounted for the association between EC and this concentration symptom in our analysis, it is possible that the symptom pertaining to trouble concentrating on things itself a manifestation of poor EC.

### Strengths and limitations

The major strength of the present study is that this is the first study to identify bridge symptoms of IA and depression networks using network analysis in a large sample of middle school students. Adequate sample size allowed us to separate data by sex and carefully examine network structures and bridge symptoms. Despite the study’s strengths, the present study has several limitations. First, the present study was conducted among middle school students in a city in Japan, limiting the generalizability of our findings in clinical samples, such as adolescents with a clinical diagnosis of major depressive disorder and pathological internet use. Given the high prevalence of subthreshold depression that does not meet the criteria of major depressive disorder yet leads to a significant health risk in adolescence (36), however, research findings in non-clinical samples can be as important as those in clinical samples in improving the well-being of adolescents. Second, the current study sample, drawn exclusively from middle school students in one city, may not be representative of the broader population of same-age students across Japan. Third, cross-sectional data limit the statements related to temporal causality between IA and depression. Future research can employ longitudinal data, including time-series data, to clarify the mechanism accounting for co-occurring IA and depression. Fourth, besides age (grade) and sex, we did not obtain data on socioeconomic status in the present study due to the constraints of the school survey. Fifth, the PHQ-A has not been validated in Japanese adolescents yet.

## Conclusion

Utilizing network analysis on cross-sectional data from a community sample of 7th to 9th-grade students, we successfully identified differences in some bridge symptoms linking IA and depression across male students and female students (“Psychomotor” in male students and “Guilty feeling” and “Functional impairment” in female students). These findings provide valuable insights for future research endeavors aimed at comprehensively understanding sex differences in the onset and persistence of comorbidity between these two conditions. Future research using a longitudinal study design is warranted to identify the directionality between effortful control and bridge symptoms and examine the role of their relationships in addressing comorbid disorders.

## Data Availability

The data analyzed in this study is subject to the following licenses/restrictions: parents of the participants in the study gave consent to the use of data only for publication and presentation but not for being shared publically. Requests to access these datasets should be directed to tomoya.hirota@ucsf.edu.

## References

[1] Kuss DJ Lopez-Fernandez O. Internet addiction and problematic Internet use: A systematic review of clinical research. World J Psychiatry. (2016) 6:143–76. doi: 10.5498/wjp.v6.i1.143 PMC4804263 27014605

[2] Ko CH Yen JY Yen CF Chen CS Chen CC. The association between Internet addiction and psychiatric disorder: a review of the literature. Eur Psychiatry. (2012) 27(1):1–8. doi: 10.1016/j.eurpsy.2010.04.011 22153731

[3] Restrepo A Scheininger T Clucas J Alexander L Salum GA Georgiades K Milham MP. Problematic internet use in children and adolescents: associations with psychiatric disorders and impairment. BMC Psychiatry. (2020) 20(1):252. doi: 10.1186/s12888-020-02640-x 32456610 PMC7251845

[4] Brunso-Bechtold JK Casagrande VA. Presence of retinogeniculate fibers is essential for initiating the formation of each interlaminar space in the lateral geniculate nucleus. Brain Res. (1985) 352:123–6. doi: 10.1016/0165-3806(85)90094-x 4005614

[5] Carli V Durkee T Wasserman D Hadlaczky G Despalins R Kramarz E. The association between pathological internet use and comorbid psychopathology: a systematic review. Psychopathology. (2013) 46:1–13. doi: 10.1159/000337971 22854219

[6] Cramer AO Waldorp LJ van der Maas HL Borsboom D. Comorbidity: a network perspective. Behav Brain Sci. (2010) 33:137–50. doi: 10.1017/s0140525x09991567 20584369

[7] van Buitenen N van den Berg CJW Meijers J Harte JM. The prevalence of mental disorders and patterns of comorbidity within a large sample of mentally ill prisoners: A network analysis. Eur Psychiatry. (2020) 63:e63. doi: 10.1192/j.eurpsy.2020.63 32522312 PMC7355171

[8] Borsboom D Deserno MK Rhemtulla M Epskamp S Fried EI McNally RJ. Network analysis of multivariate data in psychological science. Nat Rev Methods Primers. (2021) 1:58. doi: 10.1038/s43586-021-00055-w

[9] Fried EI van Borkulo CD Cramer AO Boschloo L Schoevers RA Borsboom D. Mental disorders as networks of problems: a review of recent insights. Soc Psychiatry Psychiatr Epidemiol. (2017) 52:1–10. doi: 10.1007/s00127-016-1319-z 27921134 PMC5226976

[10] Fried EI Cramer AOJ. Moving forward: challenges and directions for psychopathological network theory and methodology. Perspect Psychol Sci. (2017) 12:999–1020. doi: 10.1177/1745691617705892 28873325

[11] Jones PJ Ma R McNally RJ. Bridge centrality: A network approach to understanding comorbidity. Multivariate Behav Res. (2021) 56:353–67. doi: 10.1080/00273171.2019.1614898 31179765

[12] Cai H Bai W Sha S Zhang L Chow IHI Lei SM. Identification of central symptoms in Internet addictions and depression among adolescents in Macau: A network analysis. J Affect Disord. (2022) 302:415–23. doi: 10.1016/j.jad.2022.01.068 35065088

[13] Zhao Y Qu D Chen S Chi X. Network analysis of internet addiction and depression among Chinese college students during the COVID-19 pandemic: A longitudinal study. Comput Hum Behav. (2023) 138:107424. doi: 10.1016/j.chb.2022.107424 PMC9352366 35945974

[14] Liu S Zhang D Tian Y Xu B Wu X. Gender differences in symptom structure of adolescent problematic internet use: A network analysis. Child Adolesc Psychiatry Ment Health. (2023) 17:49. doi: 10.1186/s13034-023-00590-2 37029403 PMC10082539

[15] Smith DJ Kyle S Forty L Cooper C Walters J Russell E. Differences in depressive symptom profile between males and females. J Affect Disord. (2008) 108:279–84. doi: 10.1016/j.jad.2007.10.001 17980438

[16] Breslau J Gilman SE Stein BD Ruder T Gmelin T Miller E. Sex differences in recent first-onset depression in an epidemiological sample of adolescents. Transl Psychiatry. (2017) 7:e1139. doi: 10.1038/tp.2017.105 28556831 PMC5534940

[17] Krueger RF Eaton NR. Transdiagnostic factors of mental disorders. World Psychiatry. (2015) 14:27–9. doi: 10.1002/wps.20175 PMC4329885 25655146

[18] Rothbart MK Rueda MR. The Development of Effortful Control. In: Developing individuality in the human brain: A tribute to Michael I. Posner. American Psychological Association, Washington, DC, US (2005). p. 167–88.

[19] Karoly P. Tracking the leading edge of self-regulatory failure: commentary on “Where do we go from here? The goal perspective in psychotherapy. Clin Psychology: Sci Pract. (2006) 13:366–70. doi: 10.1111/j.1468-2850.2006.00049.x

[20] Santens E Claes L Dierckx E Dom G. Effortful control – A transdiagnostic dimension underlying internalizing and externalizing psychopathology. Neuropsychobiology. (2020) 79:255–69. doi: 10.1159/000506134 32106115

[21] Grote NK Frank E. Difficult-to-treat depression: the role of contexts and comorbidities. Biol Psychiatry. (2003) 53:660–70. doi: 10.1016/s0006-3223(03)00006-4 12706952

[22] Young KS. Internet addiction: the emergence of a new clinical disorder. CyberPsychology Behav. (1998) 1:237–44. doi: 10.1089/cpb.1998.1.237

[23] Spitzer RL Kroenke K Williams JB. Validation and utility of a self-report version of PRIME-MD: the PHQ primary care study. Primary Care Evaluation of Mental Disorders. Patient Health Questionnaire. JAMA. (1999) 282:1737–44. doi: 10.1001/jama.282.18.1737 10568646

[24] Ellis LK Rothbart M. Revision of the early adolescent temperament questionnaire. In: Poster presented at the 2001 Biennial Meeting of the Society for Research in Child Development. Society of Research in Child Development, Minneapolis, MN (2001). doi: 10.1037/t07624-000

[25] Haslbeck JMB Waldorp LJ. mgm: estimating time-varying mixed graphical models in high-dimensional data. J Stat Software. (2020) 93:1–46. doi: 10.18637/jss.v093.i08

[26] Chen J Chen Z. Extended Bayesian information criteria for model selection with large model spaces. Biometrika. (2008) 95:759–71. doi: 10.1093/biomet/asn034

[27] Fruchterman TMJ Reingold EM. Graph drawing by force-directed placement. Software: Pract Exp. (1991) 21:1129–64. doi: 10.1002/spe.4380211102

[28] Epskamp S Rhemtulla M Borsboom D. Generalized network psychometrics: combining network and latent variable models. Psychometrika. (2017) 82:904–27. doi: 10.1007/s11336-017-9557-x 28290111

[29] Epskamp S Borsboom D Fried EI. Estimating psychological networks and their accuracy: A tutorial paper. Behav Res Methods. (2018) 50:195–212. doi: 10.3758/s13428-017-0862-1 28342071 PMC5809547

[30] van Borkulo CD van Bork R Boschloo L Kossakowski JJ Tio P Schoevers RA. Comparing network structures on three aspects: A permutation test. Psychol Methods. (2023) 28:1273–85. doi: 10.1037/met0000476 35404628

[31] Borsboom D. A network theory of mental disorders. World Psychiatry. (2017) 16:5–13. doi: 10.1002/wps.20375 28127906 PMC5269502

[32] Xie Y Tang L. The symptom network of internet gaming addiction, depression, and anxiety among children and adolescents. Sci Rep. (2024) 14:29732. doi: 10.1038/s41598-024-81094-7 39614079 PMC11607465

[33] Wei X An F Liu C Li K Wu L Ren L. Escaping negative moods and concentration problems play bridge roles in the symptom network of problematic smartphone use and depression. Front Public Health. (2023) 10:981136. doi: 10.3389/fpubh.2022.981136 36733277 PMC9886682

[34] Su W Han X Jin C Yan Y Potenza MN. Are males more likely to be addicted to the internet than females? A meta-analysis involving 34 global jurisdictions. Comput Hum Behav. (2019) 99:86–100. doi: 10.1016/j.chb.2019.04.021

[35] Pandey A Hale D Das S Goddings AL Blakemore SJ Viner RM. Effectiveness of universal self-regulation-based interventions in children and adolescents: A systematic review and meta-analysis. JAMA Pediatr. (2018) 172:566–75. doi: 10.1001/jamapediatrics.2018.0232 PMC6059379 29710097

[36] Bertha EA Balázs J. Subthreshold depression in adolescence: a systematic review. Eur Child Adolesc Psychiatry. (2013) 22:589–603. doi: 10.1007/s00787-013-0411-0 23579389

