# Supplementary material for: Examining the comorbidity network of Internet addiction and depression: the role of effortful control on their bridge symptoms in adolescents
Source: Front Psychiatry. 2025 Feb 13;16:1493888. doi: 10.3389/fpsyt.2025.1493888 (PMC11866057; doi:10.3389/fpsyt.2025.1493888)
Supplement: Supplementary file 1 [file SupplementaryFile1.docx]

**Supplementary material 1: Edge strengths in the network of depression and internet addiction**

|  | Male | Female |
| --- | --- | --- |
| PHQ1-PHQ2 | 0.16 | 0.16 |
| PHQ1-PHQ3 | 0.05 | 0 |
| PHQ1-PHQ4 | 0 | 0.07 |
| PHQ1-PHQ5 | 0.17 | 0.21 |
| PHQ1-PHQ6 | 0.20 | 0.17 |
| PHQ1-PHQ7 | 0 | 0 |
| PHQ1-PHQ8 | 0.06 | 0.05 |
| PHQ1-PHQ9 | 0.25 | 0.19 |
| PHQ1-YDQ1 | 0 | 0 |
| PHQ1-YDQ2 | 0 | 0 |
| PHQ1-YDQ3 | 0 | 0 |
| PHQ1-YDQ4 | 0 | 0 |
| PHQ1-YDQ5 | 0 | 0 |
| PHQ1-YDQ6 | 0.05 | 0 |
| PHQ1-YDQ7 | 0 | 0 |
| PHQ1-YDQ8 | 0.12 | 0.14 |
| PHQ2-PHQ3 | 0.05 | 0.03 |
| PHQ2-PHQ4 | 0.01 | 0.08 |
| PHQ2-PHQ5 | 0.16 | 0.14 |
| PHQ2-PHQ6 | 0.03 | 0.04 |
| PHQ2-PHQ7 | 0.13 | 0.10 |
| PHQ2-PHQ8 | 0.09 | 0.06 |
| PHQ2-PHQ9 | 0.04 | 0.12 |
| PHQ2-YDQ1 | 0 | 0 |
| PHQ2-YDQ2 | 0 | 0 |
| PHQ2-YDQ3 | 0 | 0 |
| PHQ2-YDQ4 | 0 | 0 |
| PHQ2-YDQ5 | 0 | 0 |
| PHQ2-YDQ6 | 0 | 0 |
| PHQ2-YDQ7 | 0 | 0 |
| PHQ2-YDQ8 | 0 | 0.03 |
| PHQ3-PHQ4 | 0.23 | 0.22 |
| PHQ3-PHQ5 | 0.10 | 0.15 |
| PHQ3-PHQ6 | 0 | 0.03 |
| PHQ3-PHQ7 | 0.03 | 0.03 |
| PHQ3-PHQ8 | 0.07 | 0.08 |
| PHQ3-PHQ9 | 0 | 0.03 |
| PHQ3-YDQ1 | 0.05 | 0 |
| PHQ3-YDQ2 | 0.05 | 0.09 |
| PHQ3-YDQ3 | 0 | 0 |
| PHQ3-YDQ4 | 0 | 0.11 |
| PHQ3-YDQ5 | 0.08 | 0 |
|  | Male | Female |
| PHQ3-YDQ6 | 0 | 0 |
| PHQ3-YDQ7 | 0 | 0.09 |
| PHQ3-YDQ8 | 0.04 | 0 |
| PHQ4-PHQ5 | 0.13 | 0.13 |
| PHQ4-PHQ6 | 0.06 | 0.08 |
| PHQ4-PHQ7 | 0.07 | 0.03 |
| PHQ4-PHQ8 | 0.15 | 0.07 |
| PHQ4-PHQ9 | 0.02 | 0.04 |
| PHQ4-YDQ1 | 0 | 0 |
| PHQ4-YDQ2 | 0 | 0.08 |
| PHQ4-YDQ3 | 0.05 | 0 |
| PHQ4-YDQ4 | 0 | 0 |
| PHQ4-YDQ5 | 0 | 0 |
| PHQ4-YDQ6 | 0.08 | 0 |
| PHQ4-YDQ7 | 0 | 0 |
| PHQ4-YDQ8 | 0 | 0 |
| PHQ5-PHQ6 | 0.19 | 0.13 |
| PHQ5-PHQ7 | 0.06 | 0.07 |
| PHQ5-PHQ8 | 0.02 | 0 |
| PHQ5-PHQ9 | 0 | 0 |
| PHQ5-YDQ1 | 0 | 0 |
| PHQ5-YDQ2 | 0 | 0 |
| PHQ5-YDQ3 | 0 | 0 |
| PHQ5-YDQ4 | 0 | 0.07 |
| PHQ5-YDQ5 | 0.08 | 0.11 |
| PHQ5-YDQ6 | 0 | 0.07 |
| PHQ5-YDQ7 | 0 | 0 |
| PHQ5-YDQ8 | 0.10 | 0.19 |
| PHQ6-PHQ7 | 0.08 | 0.07 |
| PHQ6-PHQ8 | 0.09 | 0.10 |
| PHQ6-PHQ9 | 0.29 | 0.36 |
| PHQ6-YDQ1 | 0 | 0 |
| PHQ6-YDQ2 | 0 | 0.06 |
| PHQ6-YDQ3 | 0 | 0 |
| PHQ6-YDQ4 | 0 | 0 |
| PHQ6-YDQ5 | 0 | 0 |
| PHQ6-YDQ6 | 0.08 | 0.13 |
| PHQ6-YDQ7 | 0.05 | 0 |
| PHQ6-YDQ8 | 0 | 0.06 |
| PHQ7-PHQ8 | 0.19 | 0.19 |
| PHQ7-PHQ9 | 0 | 0.04 |
| PHQ7-YDQ1 | 0.08 | 0.07 |
| PHQ7-YDQ2 | 0 | 0 |
| PHQ7-YDQ3 | 0.09 | 0.11 |
| PHQ7-YDQ4 | 0 | 0.08 |
|  | Male | Female |
| PHQ7-YDQ5 | 0 | 0 |
| PHQ7-YDQ6 | 0 | 0 |
| PHQ7-YDQ7 | 0 | 0.07 |
| PHQ7-YDQ8 | 0 | 0 |
| PHQ8-PHQ9 | 0.10 | 0.03 |
| PHQ8-YDQ1 | 0 | 0 |
| PHQ8-YDQ2 | 0 | 0 |
| PHQ8-YDQ3 | 0 | 0 |
| PHQ8-YDQ4 | 0.18 | 0.11 |
| PHQ8-YDQ5 | 0 | 0 |
| PHQ8-YDQ6 | 0 | 0.10 |
| PHQ8-YDQ7 | 0 | 0 |
| PHQ8-YDQ8 | 0.05 | 0 |
| PHQ9-YDQ1 | 0 | 0 |
| PHQ9-YDQ2 | 0.11 | 0 |
| PHQ9-YDQ3 | 0 | 0 |
| PHQ9-YDQ4 | 0 | 0 |
| PHQ9-YDQ5 | 0 | 0 |
| PHQ9-YDQ6 | 0 | 0 |
| PHQ9-YDQ7 | 0 | 0 |
| PHQ9-YDQ8 | 0 | 0 |
| YDQ1-YDQ2 | 0.77 | 0.84 |
| YDQ1-YDQ3 | 0.27 | 0.29 |
| YDQ1-YDQ4 | 0.43 | 0.57 |
| YDQ1-YDQ5 | 0.17 | 0.12 |
| YDQ1-YDQ6 | 0.20 | 0 |
| YDQ1-YDQ7 | 0.13 | 0.22 |
| YDQ1-YDQ8 | 0.21 | 0.21 |
| YDQ2-YDQ3 | 0.38 | 0.33 |
| YDQ2-YDQ4 | 1.05 | 0.92 |
| YDQ2-YDQ5 | 0.36 | 0 |
| YDQ2-YDQ6 | 0.20 | 0.53 |
| YDQ2-YDQ7 | 0.14 | 0.21 |
| YDQ2-YDQ8 | 0.16 | 0 |
| YDQ3-YDQ4 | 0.34 | 0.35 |
| YDQ3-YDQ5 | 0.80 | 0.99 |
| YDQ3-YDQ6 | 0.23 | 0 |
| YDQ3-YDQ7 | 0.25 | 0.23 |
| YDQ3-YDQ8 | 0.14 | 0.24 |
| YDQ4-YDQ5 | 0 | 0 |
| YDQ4-YDQ6 | 0.60 | 0.38 |
| YDQ4-YDQ7 | 0 | 0 |
| YDQ4-YDQ8 | 0.30 | 0.40 |
| YDQ5-YDQ6 | 0 | 0 |
| YDQ5-YDQ7 | 0.49 | 0.55 |
|  | Male | Female |
| YDQ5-YDQ8 | 0.26 | 0.37 |
| YDQ6-YDQ7 | 0.57 | 0.59 |
| YDQ6-YDQ8 | 0.18 | 0 |
| YDQ7-YDQ8 | 0.25 | 0.17 |

**Supplementary material 2 (S2): Bootstrapped 95% confidence intervals of edge weights**

**Male students**

The panel above presents edge weights of the estimated network of depression and internet addiction items, and 95% confidence intervals (CIs) calculated using bootstrapping. The red line represents the original sample values, the black dots represent the bootstrap means, and the gray areas indicate the bootstrapped CIs. Each horizontal line represents one edge of the network, ordered from the edge with the highest edge weight to the edge with the lowest edge weight. The y-axis labels were removed to avoid cluttering for the simplicity of the graph.

The figure indicates that many edge weights likely do not significantly differ from one another. The large bootstrapped Cis, represented by wider gray areas, imply that interpreting the order of most edges in the network should be done with care.

**S2.**

**Female students**

**Supplementary material 3 (S3): Edge difference test**

**Male students**

Figure: In this graph, each point on the x and y axes represents a pair of edges identified in a given network. Black boxes indicate significant differences between two edges (a bootstrap stability difference test: alpha = 0.05), whereas gray boxes do not indicate any significant differences. The diagonal represents the edge strength, where white indicates weak edge strengths, while blue indicates strong edge strengths. Edge labels are omitted in this figure to keep the figure legible.

**S3.**

**Female students**

**Supplementary material 4 (S4): Centrality (expected influence) difference test**

**Male students**

**Figure:** Black boxes indicate significant differences between the centrality of two nodes (a bootstrap stability difference test: alpha = 0.05), whereas gray boxes do not indicate any significant differences. The diagonal represents the strength.

**S4. Female students**

**Supplementary material 5 (S5): Stability of network bridge centrality**

**Male students**

The figure above presents the centrality stability as assessed using the case-dropping bootstrap method. Stability was assessed by re-estimating the network based on increasingly smaller subsets of the original sample.

**S5.**

**Female students**

**Supplementary material 6 (S6): Bootstrapped 95% confidence intervals of bridge expected influence**

**Male students**

**S6: Bootstrapped 95% confidence intervals of bridge expected influence**

**Female students**

**Supplementary material 7: Edge strengths of Internet addiction and depressive symptoms with effortful control**

**Edge strengths with the total score of effortful control (EC)**

|  | Male | Female |
| --- | --- | --- |
| PHQ1 | 0 | 0 |
| PHQ2 | 0.06 | 0.05 |
| PHQ3 | 0.05 | 0.03 |
| PHQ4 | 0.06 | 0.06 |
| PHQ5 | 0 | 0.06 |
| PHQ6 | 0.11 | 0.07 |
| PHQ7 | 0.21 | 0.22 |
| PHQ8 | 0.05 | 0.1 |
| PHQ9 | 0 | 0 |
| YDQ1 | 0.1 | 0.22 |
| YDQ2 | 0 | 0 |
| YDQ3 | 0.19 | 0.2 |
| YDQ4 | 0 | 0.12 |
| YDQ5 | 0.2 | 0.23 |
| YDQ6 | 0 | 0.1 |
| YDQ7 | 0.18 | 0.19 |
| YDQ8 | 0.12 | 0 |

**Edge strengths with the EC subdomain score (activation control)**

|  | Male | Female |
| --- | --- | --- |
| PHQ1 | 0 | 0 |
| PHQ2 | 0 | 0.07 |
| PHQ3 | 0 | 0.04 |
| PHQ4 | 0 | 0 |
| PHQ5 | 0 | 0 |
| PHQ6 | 0.05 | 0.03 |
| PHQ7 | 0.06 | 0 |
| PHQ8 | 0 | 0 |
| PHQ9 | 0 | 0 |
| YDQ1 | 0.08 | 0.06 |
| YDQ2 | 0 | 0 |
| YDQ3 | 0 | 0.14 |
| YDQ4 | 0 | 0.10 |
| YDQ5 | 0.13 | 0.13 |
| YDQ6 | 0 | 0 |
| YDQ7 | 0.08 | 0 |
| YDQ8 | 0.04 | 0 |

**S6. Edge strengths with the EC subdomain score (inhibitory control)**

|  | Male | Female |
| --- | --- | --- |
| PHQ1 | 0 | 0 |
| PHQ2 | 0 | 0 |
| PHQ3 | 0 | 0 |
| PHQ4 | 0 | 0 |
| PHQ5 | 0 | 0 |
| PHQ6 | 0 | 0 |
| PHQ7 | 0 | 0 |
| PHQ8 | 0.03 | 0 |
| PHQ9 | 0 | 0 |
| YDQ1 | 0 | 0 |
| YDQ2 | 0 | 0 |
| YDQ3 | 0 | 0 |
| YDQ4 | 0 | 0 |
| YDQ5 | 0 | 0 |
| YDQ6 | 0.13 | 0 |
| YDQ7 | 0 | 0 |
| YDQ8 | 0 | 0 |

**Edge strengths with the EC subdomain score (attention)**

|  | Male | Female |
| --- | --- | --- |
| PHQ1 | 0 | 0 |
| PHQ2 | 0 | 0 |
| PHQ3 | 0 | 0 |
| PHQ4 | 0 | 0 |
| PHQ5 | 0 | 0.06 |
| PHQ6 | 0 | 0 |
| PHQ7 | 0.13 | 0.20 |
| PHQ8 | 0.06 | 0 |
| PHQ9 | 0 | 0 |
| YDQ1 | 0 | 0 |
| YDQ2 | 0 | 0 |
| YDQ3 | 0 | 0 |
| YDQ4 | 0.13 | 0 |
| YDQ5 | 0 | 0.05 |
| YDQ6 | 0 | 0 |
| YDQ7 | 0 | 0 |
| YDQ8 | 0 | 0 |
